# Association of Admission Functional Status and Body Mass Index with Mortality in Patients Receiving Chronic Dialysis: A Nationwide Observational Cohort Study

**DOI:** 10.31662/jmaj.2022-0188

**Published:** 2023-10-04

**Authors:** Shintaro Mandai, Takaaki Koide, Tamami Fujiki, Yutaro Mori, Fumiaki Ando, Koichiro Susa, Takayasu Mori, Soichiro Iimori, Shotaro Naito, Eisei Sohara, Shinichi Uchida, Kiyohide Fushimi, Tatemitsu Rai

**Affiliations:** 1 Department of Nephrology, Graduate School of Medical and Dental Sciences, Tokyo Medical and Dental University, Tokyo, Japan; 2 Department of Health Policy and Informatics, Graduate School of Medical and Dental Sciences, Tokyo Medical and Dental University, Tokyo, Japan; 3 Department of Nephrology and Hypertension, Dokkyo Medical University, Shimotsuga, Japan

**Keywords:** activity of daily living, functional status, age, body mass index, dialysis, chronic kidney disease

## Abstract

**Introduction:**

Chronic kidney disease (CKD) significantly affects activities of daily living (ADLs) before and after the initiation of dialysis, particularly in elderly individuals. However, the impact of admission functional status on dialysis patients’ outcome is not fully understood. This study aimed to investigate the effect of the number of ADL disabilities usually measured for all patients hospitalized in Japan on in-hospital outcome for dialysis patients.

**Methods:**

Using an inpatient administrative claims database, we included 104,557 admissions of patients undergoing chronic dialysis aged 65 years and above from 2012 to 2014. The primary outcome was in-hospital all-cause mortality (evaluated using logistic regression models), and the secondary outcomes were length of stay and care cost.

**Results:**

The mean age of the participants was 74.0 ± 6.2 years, the mean body mass index (BMI) was 21.8 ± 3.9, 31% needed assistance for one or more of five basic ADLs (feeding, transferring, going to toilet, dressing, and bathing) at admission, and 3.5% (n = 3,701) died after hospitalization. After adjusting for confounding factors, the odds ratios (ORs) (95% confidence intervals) of death for 1, 2, 3, 4, and 5 ADL disabilities were 1.43 (1.19-1.70), 2.04 (1.71-2.45), 2.58 (2.19-3.04), 3.74 (3.35-4.17), and 6.83 (6.29-7.41) versus a complete independence, respectively. The increasing number of ADL disabilities was also associated with greater length of stay and costs. Risk stratification by age, admission functional status, and BMI showed an 18-mortality risk matrix with a maximal risk of a 15.5-higher OR for lean patients aged ≥75 years with severe ADL disability compared with that for patients aged <75 years with middle BMI and no ADL disability on admission.

**Conclusions:**

Admission functional status decline significantly increases in-hospital mortality, length of stay, and costs. Routine assessment of functional status can facilitate the risk prediction of dialysis patients.

## Introduction

Chronic kidney disease (CKD) affects 700 million people globally ^(1)^, and progression to end-stage kidney disease (ESKD) requires kidney replacement therapy. CKD and ESKD lead to high morbidity and mortality rates, thus representing social and economic burden with the increasing number of affected aged patients ^(1), (2)^. Numerous studies have demonstrated that CKD significantly affects activities of daily living (ADLs) due to sarcopenia and multiple comorbidities ^(3), (4)^. Furthermore, the functional status of elderly patients declines even after the dialysis initiation ^(5)^.

The global prevalence of sarcopenia has been rising among the aging population ^(6)^. The severity of sarcopenia progresses with age and as kidney function declines ^(7), (8)^. Muscle weakness and ADL impairment are known to be associated with increased mortality ^(9), (10)^, whereas a functional status decline in the long-term prognosis has been recently reported among patients with CKD and undergoing dialysis ^(3), (11)^. However, the relationship between ADLs on admission and in-hospital mortality is not fully understood.

Mortality rates are high among chronic dialysis patients despite predialysis and dialysis care advancements. The risk of in-hospital mortality among elderly patients is significantly higher than that in the general population ^(12), (13), (14)^. We hypothesized that admission functional status determines the in-hospital outcome of dialysis patients. Our study aimed to investigate the association of five basic ADL (feeding, transferring, going to toilet, dressing, and bathing) disabilities routinely assessed at Japanese hospitals for mortality, hospital length of stay, and costs. We focused on the ADLs of patients with a planned hospital admission, who are more likely to be correctly examined. We also determined whether the underlying cause for admission or the patients’ body mass index (BMI) modifies the impact of ADLs on outcomes.

We found that the in-hospital mortality rate among dialysis patients was very high, even in planned admissions. The increasing number of ADL disabilities was strongly associated with the outcome, cost, and length of hospital stay. This study provides clinical implications as a predictor and the importance of usual care of ADLs to improve in-hospital outcomes and survival of dialysis patients.

## Materials and Methods

### Source of data

The study cohort was obtained from the Diagnosis Procedure Combination (DPC) of the Japanese administrative claims database ^(15), (16)^. DPC is a case-mix classification system linked with a payment system that includes all 82 university teaching hospitals. Thus, this database is based on cases from more than 1,000 hospitals and includes >50% of cases in Japan. It contains the unique hospital identifying number; patient age and sex; five basic ADLs (feeding, transferring, going to the toilet, dressing, and bathing); dialysis modality (hemodialysis, peritoneal dialysis, or both); final discharge diagnoses and comorbidities at admission coded according to the International Classification of Diseases and Related Health Problems, 10^th^ Revision (ICD-10) ^(17)^; and the Charlson Comorbidity Index ^(18)^ updated for use in patients with ESKD ^(15), (19)^ at admission. The database also included information on patient care processes, drug administration, or devices used.

This study was conducted in accordance with the ethical principles of the Declaration of Helsinki and approved by the ethics committee of Tokyo Medical and Dental University (No. M2000-788). The requirement for informed consent was waived due to the anonymous nature of the data.

### Patient selection and study design

This is a retrospective cohort study involving patients aged ≥65 years who had undergone chronic dialysis during hospitalization (Figure S1). In total, there were 267,976 admissions between 2012 and 2014. We excluded emergent admissions (due to the difficulty in accurately assessing the functional status), nonelective admissions, vascular access-related admissions, and admissions without information on functional abilities, BMI, or admission type (emergent or elective admission). Overall, 104,557 admissions were included in the final analysis (Figure S1).

Patients undergoing maintenance hemodialysis and peritoneal dialysis were identified based on the coding of patient care procedures as follows: chronic maintenance hemodialysis lasting <4, 4-5, or ≥5 h per session or chronic maintenance hemodiafiltration or continuous peritoneal dialysis ^(15)^.

### Patient characteristics and assessment of admission functional status

In the database, five basic ADLs (feeding, transferring, going to toilet, dressing, and bathing) at admission were identified ^(20)^. Independence in ADLs was defined as requiring partial or complete assistance in feeding, transferring, going to toilet, or dressing. According to the database, independence for bathing was described as requiring complete assistance in bathing. Other admission data included age, sex, BMI, dialysis modality (hemodialysis, peritoneal dialysis, or both), Charlson Comorbidity Index scores updated for use in patients with ESKD ^(15), (19)^, type (emergency or elective admission), and fiscal year of admission.

### Outcome

**Figure 1.**
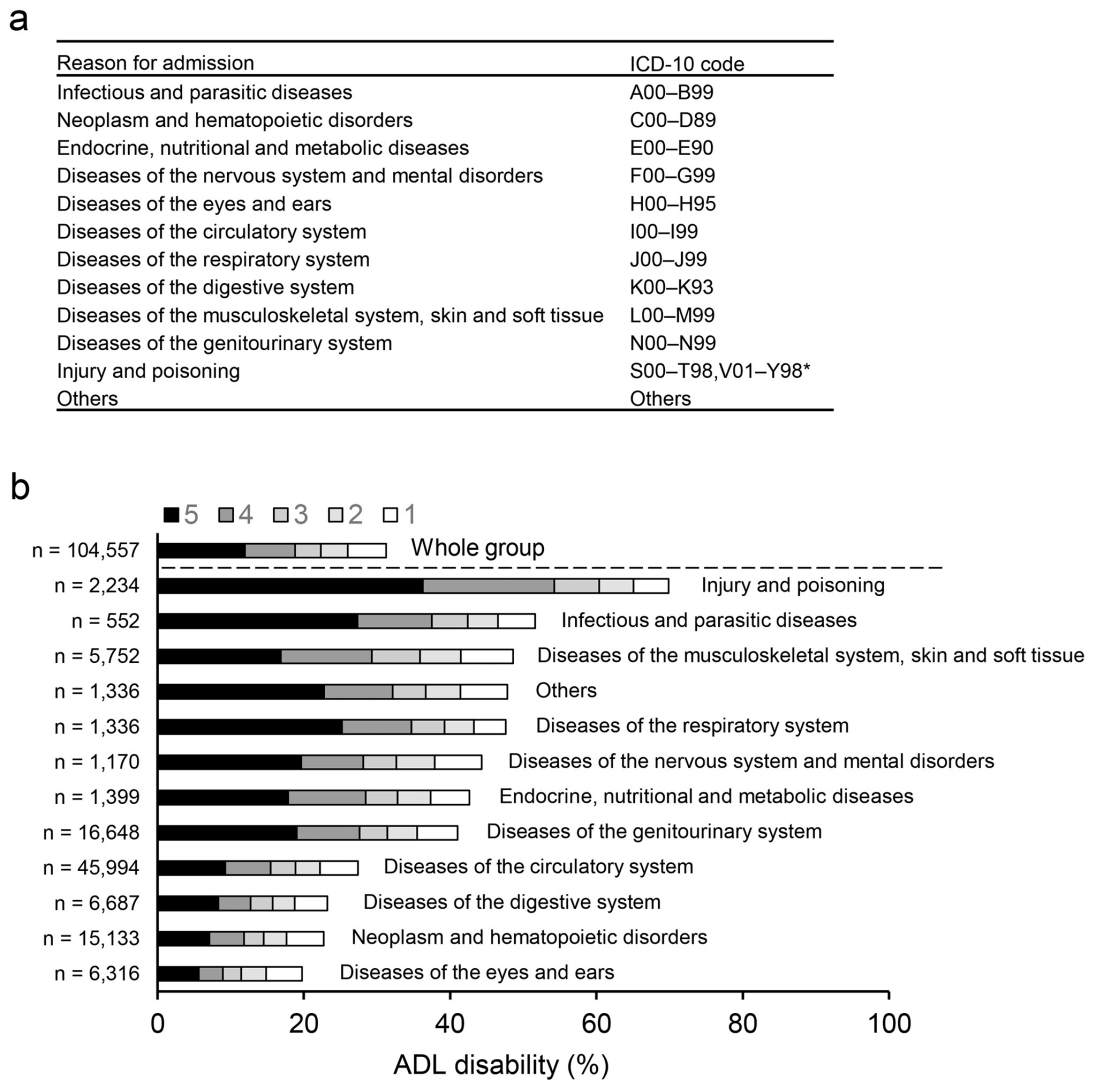
Rates of dependence of basic ADLs at admission according to the reasons for admission among maintenance dialysis patients. a, The diagnostic causes of hospitalization were stratified into the 12 categories shown based on the International Classification of Diseases and Related Health Problems, 10th Revision (ICD-10). b, Proportion of each number that required assistance for the five basic ADLs (feeding, transferring, toileting, dressing, and bathing) according to the diagnostic causes of hospitalization in end-stage kidney disease.

The primary outcome was mortality during hospitalization. The diagnostic causes of hospitalization were stratified into the following 12 categories based on ICD-10 codes ^(17)^ (Figure 1a): 1) infectious and parasitic diseases (n = 552); 2) neoplasm and hematopoietic disorders (n = 15,133); 3) endocrine, nutritional, and metabolic diseases (n = 1,399); 4) diseases of the nervous system and mental disorders (n = 1,170); 5) diseases of the eyes and ears (n = 6,316); 6) diseases of the circulatory system (n = 45,994); 7) diseases of the respiratory system (n = 1,336); 8) diseases of the digestive system (n = 6,687); 9) diseases of the musculoskeletal system, skin, and soft tissue (n = 5,752); 10) diseases of the genitourinary system (n = 16,648); 11) injury and poisoning (n = 2,234); and 12) others (n = 1,336). The secondary outcomes were hospital length of stay and inpatient care costs. The total costs during hospitalization were calculated according to reference prices in the fee schedule that determine item-by-item prices.

### Data analyses

Baseline characteristics are expressed as mode (n, percentage) or mean (standard deviation). Comparisons among the multiple groups were evaluated using the Wilcoxon test for continuous variables and chi-squared test for categorical variables. We conducted logistic regression analyses to examine factors associated with in-hospital mortality, length of stay ≥30 days, or medical costs ≥highest quartile in dialysis patients. This model was adjusted for age, sex, BMI, dialysis modality, and Charlson Comorbidity Index score. Statistical analyses were conducted using JMP Pro 12.0 (SAS Institute Inc., Cary, USA) and Stata version 15.0 (Stata Corp., College Station, TX, USA). *P* values < 0.05 were considered statistically significant.

## Results

### Patient characteristics and prevalence of functional disabilities

Our study included 104,557 patients with chronic dialysis admitted between 2012 and 2014 in Japan (Figure S1). The characteristics according to the number of ADL disabilities are presented in Table 1. Our study included patients with a mean age of 74.0 ± 6.2 years and a mean BMI of 21.8 ± 3.9 kg per m^2^; 31.2% of them were women, and 94.6% were dependent on hemodialysis. As the number of ADL disabilities increased, the age of patients, prevalence of women, lean body mass, and dependence on hemodialysis increased. Patients with lower ADLs were more likely to have various comorbidities, including peripheral vascular disease, cerebrovascular disease, dementia, chronic pulmonary disease, rheumatologic disease, diabetes mellitus, and solid metastatic tumor, leading to higher Charlson Comorbidity Index scores.

**Table 1.** Characteristics of Elderly Dialysis Patients by ADL Strata from the Japanese National Inpatient Database 2012-2014.

		Number of ADL disability						
	Whole group	0	1	2	3	4	5	*P* value
N	104,557	71,866	5,531	3,801	3,727	7,156	12,476	
Age (yr)	74.0 ± 6.2	73.1 ± 5.8	75.0 ± 6.4	75.0 ± 6.5	75.4 ± 6.5	76.1 ± 6.6	76.7 ± 6.8	<0.0001
Female (%)	31.2	27.6	36.1	40.0	38.4	38.6	40.8	<0.0001
BMI (kg per m^2^)	21.8 ± 3.9	22.1 ± 3.8	21.8 ± 3.7	21.8 ± 3.7	21.7 ± 3.7	21.4 ± 3.7	20.7 ± 4.1	<0.0001
Dialysis modality (%)								
HD	94.6	93.8	95.4	95.8	96.8	96.9	96.3	<0.0001
PD	4.5	5.3	3.7	3.1	2.5	2.4	3.0	
HD + PD	0.9	0.9	0.9	1.1	0.7	0.7	0.7	
Charlson comorbidity (%)								
Myocardial infarction	3.2	3.2	3.2	3.1	3.3	3.1	2.9	0.7
Congestive heart failure	12.5	12.5	12.9	11.9	12.2	12.1	12.7	0.5
Peripheral vascular disease	8.4	7.7	9.5	8.9	10.6	10.7	10.0	<0.0001
Cerebrovascular disease	6.8	5.0	7.4	7.6	8.6	9.3	14.4	<0.0001
Dementia	2.1	0.9	2.3	2.6	2.7	4.1	7.5	<0.0001
Chronic pulmonary disease	1.6	1.5	1.7	1.2	1.6	2.1	1.8	0.0001
Rheumatologic disease	0.8	0.6	1.2	1.4	1.3	1.1	1.3	<0.0001
Peptic ulcer disease	2.9	3.0	2.9	2.9	3.0	2.6	2.4	0.0093
Mild liver disease	3.5	3.5	2.9	3.3	3.3	3.1	3.5	0.1
Diabetes without chronic complications	13.6	13.6	14.2	14.0	13.9	13.8	12.9	0.1
Diabetes with chronic complications	22.0	20.1	24.5	23.8	26.3	28.0	26.7	<0.0001
Hemiplegia or paraplegia	0.3	0.1	0.3	0.3	0.4	0.6	1.1	<0.0001
Any malignancy, including leukemia and lymphoma	5.9	5.9	5.8	5.5	5.6	5.8	6.0	0.8
Moderate or severe liver disease	0.2	0.2	0.2	0.2	0.2	0.2	0.3	0.2
Metastatic solid tumor	1.4	1.3	1.4	1.6	1.8	1.6	1.6	0.0019
AIDS/HIV	0.01	0.01	0	0	0	0	0.01	0.8
Charlson comorbidity index (%)								
0-1	59.9	61.8	58.1	59.2	56.8	56.4	52.6	<0.0001
2-3	32.2	31.1	33.6	32.6	34.3	34.2	36.8	
≥4	7.9	7.1	8.3	8.2	8.9	9.4	10.6

Data are percentiles or means (standard deviations). ADL, activities of daily living; BMI, body mass index; HD, hemodialysis; PD, peritoneal dialysis

Overall, 31% of the participants required assistance with one or more of the five basic ADLs. Investigating the possible reasons for admission of the patients (classified according to ICD-10 codes) (Figure 1a), injury and poisoning (70%) had the highest prevalence of ADL disabilities, followed by infectious and parasitic diseases (51%) and then musculoskeletal system diseases (49%) (Figure 1b). After admission with other disease categories, 20%-48% of the patients needed assistance with one or more ADLs.

### Factors associated with in-hospital mortality risk among maintenance dialysis patients

Overall, 3,701 (3.5%) dialysis patients died after hospitalization. We conducted multivariable logistic regression analyses to investigate factors associated with in-hospital death. After adjusting for potential confounders, the risk of mortality increased with age (Table 2). The odds ratios (ORs) for ages 75-84 years and >85 years were 1.31 (95% confidence interval [CI], 1.22-1.41) and 1.76 (95% CI, 1.57-1.97), respectively, versus that in the 65-74-year-old age group. The differences in the mortality risk between male and female patients showed a lower OR among women (0.81; 95% CI, 0.75-0.87). A lean body mass with BMI ≤18.4 affected the mortality risk, with the OR being 1.80 (95% CI, 1.66-1.97) versus that in the 18.5-24.9 BMI group. Contrarily, overweight patients with BMI ≥25.0 had a lower OR (0.89; 95% CI, 0.80-0.97). The ORs for 1, 2, 3, 4, and 5 ADL disabilities were 1.43 (95% CI, 1.19-1.70), 2.04 (95% CI, 1.71-2.45), 2.58 (95% CI, 2.19-3.04), 3.74 (95% CI, 3.35-4.17), and 6.83 (95% CI, 6.29-7.41), respectively, suggesting a strong association between admission functional status and in-hospital mortality risk among chronic dialysis patients.

**Table 2.** Factors Associated with In-Hospital Mortality in Elderly Subjects on Maintenance Dialysis.

		OR (95% CI)	
Variable	No. of Events/N	Crude OR	Adjusted OR^a^
Age (yr)			
65-74	1,511/58,742	Reference	Reference
75-84	1,701/39,427	1.71 (1.59-1.83)	1.31 (1.22-1.41)
≥85	489/6,388	3.14 (2.83-3.49)	1.76 (1.57-1.97)
Female	1,178/32,657	1.03 (0.96-1.10)	0.81 (0.75-0.87)
BMI (kg per m^2^)			
≤18.4	1,160/16,216	2.48 (2.30-2.67)	1.80 (1.66-1.94)
18.5-24.9	2,053/68,194	Reference	Reference
≥25.0	488/20,147	0.80 (0.72-0.88)	0.89 (0.80-0.97)
No. of ADL disability			
0	1,187/71,866	Reference	Reference
1	141/5,531	1.56 (1.31-1.86)	1.43 (1.19-1.70)
2	137/3,801	2.23 (1.86-2.67)	2.04 (1.71-2.45)
3	173/3,727	2.90 (2.46-3.41)	2.58 (2.19-3.04)
4	492/7,156	4.40 (3.95-4.90)	3.74 (3.35-4.17)
5	1,571/12,476	8.58 (7.93-9.27)	6.83 (6.29-7.41)

^a^Adjusted for age, sex, body mass index, No. of ADL disability, dialysis modality, and Charlson Comorbidity Index. ADL, activities of daily living; BMI, body mass index; CI, confidence interval; OR, odds ratio

Next, we determined the impact of each basic ADL disability on in-hospital mortality risk. As presented in Table S1, the adjusted ORs for disability with feeding, transferring, going to toilet, dressing, and bathing were 4.45 (95% CI, 4.14-4.77), 4.38 (95% CI, 4.08-4.70), 4.70 (95% CI, 4.38-5.04), 4.64 (95% CI, 4.32-4.98), and 4.29 (95% CI, 3.99-4.60).

**Figure 2.**
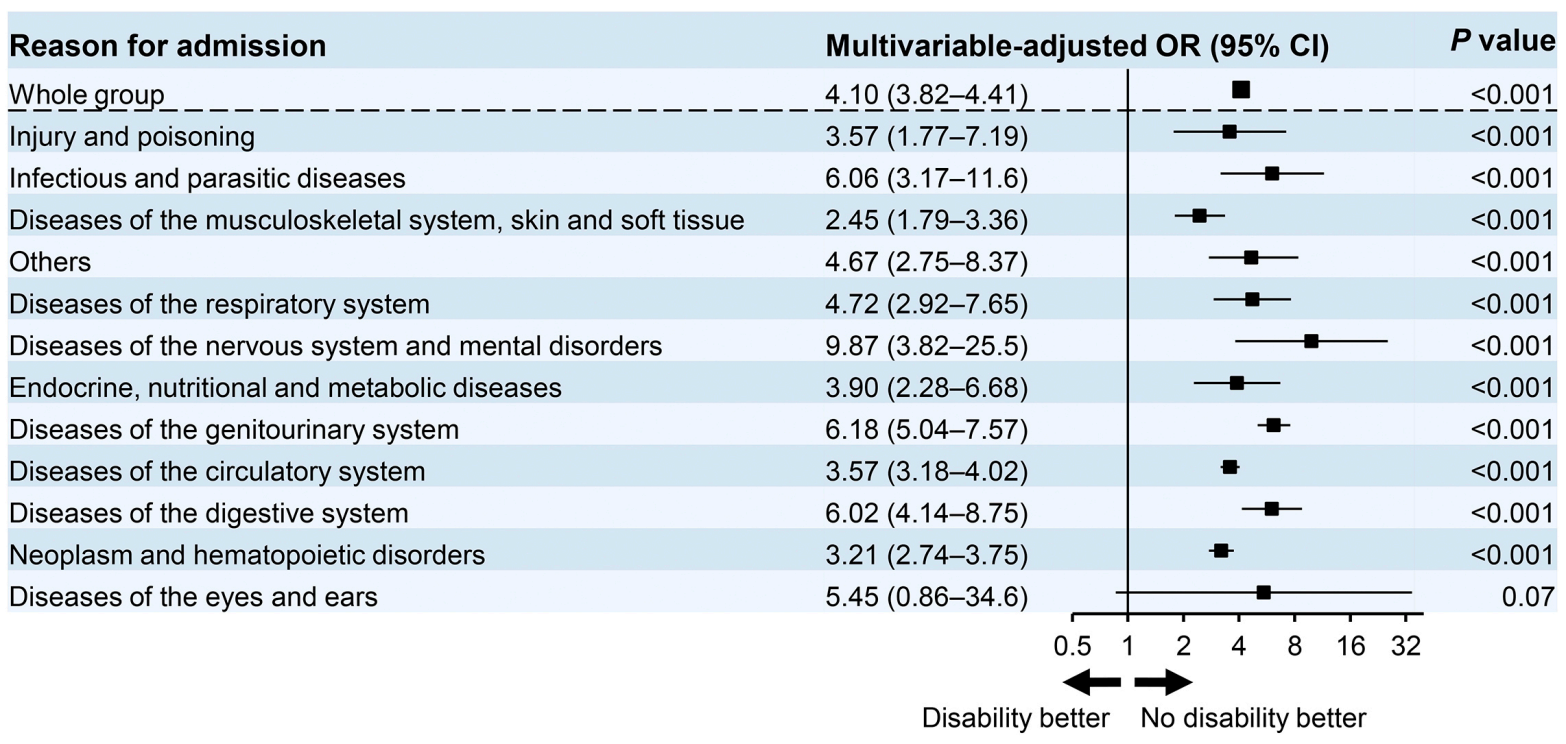
Odds ratios for in-hospital mortality according to the reason for admission in dialysis patients with ADL disability versus no disability. Models were adjusted for age, sex, body mass index, dialysis modality, and Charlson Comorbidity Index. Each box represents a point estimate of OR, and the solid lines represent the corresponding 95% CI. ADL, activities of daily living; CI, confidence interval; OR, odds ratio.

We further examined the impact of functional status decline with one or more ADL disabilities on in-hospital mortality according to the reasons for hospitalization. The logistic regression models were adjusted for age, sex, BMI, dialysis modality, and Charlson Comorbidity Index scores. A decrease in ADLs was associated with an increased risk of in-hospital mortality with an OR of 4.10 (95% CI, 3.82-4.41) (Figure 2). Among patients hospitalized for diseases of the nervous system and mental disorders, ADL disability particularly increased the in-hospital mortality risk (OR, 9.87; 95% CI, 3.82-25.5). The functional status decline at admission was associated with higher mortality independent of the reasons for admission among dialysis patients.

### Impact of admission functional status on hospital length of stay and cost

To determine the impact of ADL disability on the length of hospital stay and healthcare costs in elderly dialysis patients, we conducted logistic regression analyses with adjustments for age, sex, BMI, dialysis modality, and Charlson Comorbidity Index.

The median length of stay was 9 days (IQR, 4-23). As presented in Table 3, dependence of 1, 2, 3, 4, and 5 ADLs was associated with a higher risk for a longer stay of ≥30 days with ORs of 1.88 (95% CI, 1.76-2.02), 2.27 (95% CI, 2.10-2.02), 2.60 (95% CI, 2.41-2.80), 3.36 (95% CI, 3.19-3.55), and 4.17 (95% CI, 3.99-4.35), respectively, versus complete independence. Table S2 presents the effect of each disability type on the risk for a longer stay. The disabilities with basic ADLs were equally associated with a longer hospital stay independent of ADL types.

**Table 3.** Impact of ADL Disability on Hospital Length of Stay and Medical Cost in Elderly Dialysis Patients.

		OR (95% CI)	
Category	No. of Events/N	Crude	Adjusted^a^
Long length of stay (≥30 days)			
No. of ADL disability			
0	9,356/71,866	Reference	Reference
1	1,258/5,531	1.97 (1.84-2.10)	1.88 (1.76-2.02)
2	999/3,801	2.38 (2.21-2.57)	2.27 (2.10-2.45)
3	1,087/3,727	2.75 (2.56-2.96)	2.60 (2.41-2.80)
4	2,498/7,156	3.58 (3.40-3.78)	3.36 (3.19-3.55)
5	5,031/12,476	4.51 (4.33-4.71)	4.17 (3.99-4.35)
Highest quartile of medical cost			
No. of ADL disability			
0	16,303/71,866	Reference	Reference
1	1,457/5,531	1.22 (1.15-1.30)	1.21 (1.13-1.29)
2	1,064/3,801	1.32 (1.23-1.43)	1.31 (1.22-1.41)
3	1,076/3,727	1.38 (1.29-1.49)	1.35 (1.26-1.46)
4	2,271/7,156	1.58 (1.50-1.67)	1.56 (1.48-1.65)
5	3,968/12,476	1.59 (1.52-1.66)	1.57 (1.50-1.63)

^a^Adjusted for age, sex, body mass index, No. of ADL disability, dialysis modality, and Charlson Comorbidity Index. ADL, activities of daily living; CI, confidence interval; OR, odds ratio

The median cost was 452,580 Japanese yen (IQR, 112,140-1,120,434 yen), which is equal to 5463 US dollars (IQR, 1,354-13,524 dollars) based on the exchange rate on April 02, 2012. The number of ADL disabilities, 1, 2, 3, 4, and 5, were associated with a greater risk of medical costs ≥highest quartile with ORs of 1.21 (95% CI, 1.13-1.29), 1.31 (95% CI, 1.22-1.41), 1.35 (95% CI, 1.26-1.46), 1.56 (95% CI, 1.48-1.65), and 1.57 (95% CI, 1.50-1.63), respectively, versus the risk associated with no disabilities. As presented in Table S2, each functional disability was equally linked to increased risk of higher inpatient care costs. These findings suggest that lower ADLs at admission significantly affect length of stay and medical costs in patients on dialysis.

### Risk stratification of in-hospital death in hospitalized dialysis patients using age, admission functional status, and BMI

To establish a clinically meaningful predication model for in-hospital death in dialysis patients, we created a heat map for ORs of death using admission ADLs with a combination of age and BMI categories. ORs were determined using logistic regression models with adjustments for sex, dialysis modality, and Charlson Comorbidity Index.

**Figure 3.**
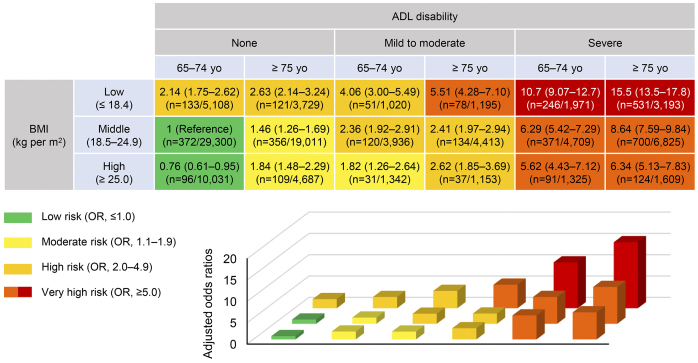
A risk matrix for in-hospital mortality according to admission functional status, age, and BMI categories. The odds ratio with 95% CI and numbers of events and individuals are described for each category. Models were adjusted for sex, dialysis modality, and Charlson Comorbidity Index. ADL, activities of daily living, BMI, body mass index.

As presented in Figure 3, we made 18 matrix categories according to age (65-74 years or ≥75 years), BMI (lean body mass, BMI ≤18.4; middle body mass, BMI 18.5-24.9; or overweight, BMI ≥25.0), and ADL disabilities (none; mild to moderate, dependence of 1-3 basic ADLs; or severe, dependence of 4 or 5 basic ADLs) and determined a risk of death for each matrix versus the patients aged <75 years with middle BMI and no ADL disability on admission. The ORs ranged from 0.76 to 15.5, as displayed in the heat map using four colors representing: OR, <2.00; OR, 2.00-4.99; OR, 5.00-9.99; and OR, ≥10.00 (Figure 3). A paradoxical association between higher BMIs and lower mortality was observed independent of ages and ADLs. More serious disabilities were significantly associated with higher risk of death particularly in higher ages and lower BMIs. Lean patients aged ≥75 years with severe ADL disability had a maximal risk of a 15.5-fold higher OR compared with that of patients aged <75 years with middle BMI and no ADL disability on admission.

These analyses were also conducted among various clinical subgroups, including male/female, diabetes mellitus/no diabetes mellitus, CVD/no CVD, or Charlson Comorbidity Index scores. As presented in Figure S2, the relationship between the risk stratification matrix and mortality risk was similar between male and female patients or diabetes mellitus and no diabetes mellitus. It is noteworthy that the effect of lower admission ADLs and BMI on higher mortality was slightly weaker for patients with underlying CVD compared with non-CVD patients. Similarly, weaker trends were observed in patients with greater comorbidity scores when compared with patients with lower scores.

## Discussion

This population-based study demonstrated that objective admission functional status significantly impacts the in-hospital mortality of chronic dialysis patients. We found that functional disability equally affects survival rates independent of the reasons for admission. Increasing numbers of functional disabilities were also associated with longer hospital stays and patient care costs. To the best of our knowledge, this is the first study to assess the impact of admission ADL decline on the risk of in-hospital mortality in elderly dialysis patients.

This study showed that the increase in admission basic functional disabilities significantly increases in-hospital mortality risk. The continuous effect on essential ADLs by aging >70-years old has long been recognized ^(21)^. Importantly, disturbance in basic ADLs decreases survival in patients with eGFR <60 mL/min/1.73 m^2^ but not in those without kidney dysfunction; furthermore, it has a greater impact on mortality than that in instrumental ADLs ^(22)^ Patients with CKD are susceptible to functional disabilities, affecting disability-free survival ^(3), (4), (5), (23)^. Recent studies found that chronic impairment of objective or self-reported basic ADLs is associated with increased mortality in patients undergoing long-term dialysis ^(20), (24)^. In this study, we focused on the previously uncovered association between admission functional status and in-hospital outcomes in ESKD. In the general population, ADLs at admission have been associated with in-hospital mortality risk for specific diseases or situations, such as a major surgery or admission to a medical intensive care unit ^(25), (26)^. To the best of our knowledge, this report is the first to show a clear association between ADLs at admission and increased risks for in-hospital mortality, longer stay, and higher costs in dialysis patients.

We found that the risk of in-hospital mortality increased with declining ADLs in dialysis patients, independent of disease categories at admission. Throughout the study group, a higher BMI was associated with improved survival, which may be called an obesity paradox. We determined whether age and BMI modify this impact of ADLs on mortality. As shown in the heat map, the risk of death increased in patients with low BMI, independent of age and ADL status, whereas the impacts of being overweight on mortality risk depended on age and ADL status. This risk stratification matrix may be useful as a predictor of in-hospital mortality and for decision-making regarding the hospitalization of dialysis patients.

Previous studies reported that in the general population, patients with low BMI have worse prognosis after hospitalization ^(26), (27)^. Furthermore, low food intake during hospitalization in the general population worsens the prognosis ^(28), (29)^. In this study, and we found that severe ADL disabilities increased the mortality rate, particularly in dialysis patients with a low BMI. A nutritional intervention might improve in-hospital outcomes for dialysis patients. Survival rates can also be improved by consuming a high-protein diet and amino acid loading during hospitalization in the general population ^(30), (31)^. Although the usual exposure to a high-protein diet may be harmful for the kidney, an intake of high-energy and high-protein diet limited to the hospitalization period may improve prognosis in acute care settings. However, BMI does not necessarily reflect lean body mass. A low BMI may be partly attributed to dehydration that can influence the study findings. Further research focusing on specific nutritional interventions (e.g., how much protein and energy are needed) is warranted to reduce in-hospital mortality among dialysis patients.

Early rehabilitation intervention for patients with low ADLs, particularly lean patients, may improve the risk of in-hospital mortality, given that low ADLs increase the risk of in-hospital mortality. Although a proportion of dialysis patients have limited physical activities due to preexisting impaired ADLs, exercise is known to improve the prognosis of these patients ^(32), (33)^. Early rehabilitation intervention has been found to improve in-hospital mortality in the general population ^(34), (35)^. Due to the lack of studies investigating whether exercise interventions improve in-hospital outcomes in dialysis patients, future interventional studies are warranted.

This study has several limitations. First, although we chose elective admissions, the patients’ ADLs may be partially affected by the cause of admissions; the severity of the disease potentially reflects the patient outcome. Second, the primary cause of death, which could explain the concise relationship between lower ADLs and poor outcome following hospitalization, was not available because of the nature of our inpatient database. Third, the dialysis vintage of the participants related to low ADLs and outcome was unavailable in our datasets. Fourth, our database also lacked laboratory data that are potential confounders, which may influence the relationship of ADLs or BMI with outcomes. Fifth, the study subjects were drawn from a single ethnicity. However, the DPC database covers approximately half of admission cases in Japan, and the population evaluated here thus represents the Japanese dialysis population ^(15)^. Finally, the follow-up data of the participants were not available in the datasets. Thus, further studies are warranted to elucidate the relationship between admission functional status and long-term outcome in patients on chronic dialysis.

In summary, admission functional status is a strong predictor of in-hospital outcome in elderly chronic dialysis patients, regardless of the disease that precipitated admission. Physicians need to be more cautious in treating dialysis patients with low ADLs and lean body mass, even in scheduled admissions.

## Article Information

### Conflicts of Interest

None

### Sources of Funding

This work was supported by The Health and Labour Sciences Research Grant (grant number. Seisaku-Sitei-22AA2003 to KF) of the Japan Ministry of Health, Labour and Welfare.

### Acknowledgement

We thank the study participants.

### Author Contributions

Shintaro Mandai: conceptualization, formal analysis, writing - original draft, writing - review and editing, project administration; Takaaki Koide: formal analysis, writing - original draft; Tamami Fujiki: formal analysis; Yutaro Mori: investigation; Fumiaki Ando: investigation; Koichiro Susa: investigation; Takayasu Mori: investigation; Soichiro Iimori: formal analysis; Shotaro Naito: formal analysis; Eisei Sohara: investigation; Shinichi Uchida: supervision; Kiyohide Fushimi: resources, supervision; Tatemitsu Rai: project administration, supervision.

Shintaro Mandai and Takaaki Koide contributed equally to this work.

### Approval by Institutional Review Board (IRB)

This study was approved by the ethics committee of Tokyo Medical and Dental University (No. M2000-788).

### Disclaimer

Shinichi Uchida is one of the Associate Editors of JMA Journal and on the journal’s Editorial Staff. He was not involved in the editorial evaluation or decision to accept this article for publication at all.

## Supplement

Supplementary Files
