# Supplementary material for: Association of Admission Functional Status and Body Mass Index with Mortality in Patients Receiving Chronic Dialysis: A Nationwide Observational Cohort Study
Source: JMA J. 2023 Oct 4;6(4):404–13. doi: 10.31662/jmaj.2022-0188 (PMC10628309; doi:10.31662/jmaj.2022-0188)
Supplement: Supplementary Files [file 2433-3298-6-4-404-s001.pdf]

Association of admission functional status and body mass index with mortality in patients receiving chronic dialysis: A nationwide observational cohort study

Shintaro Mandai[1]*, Takaaki Koide[1], Tamami Fujiki[1], Yutaro Mori[1], Fumiaki Ando[1], Koichiro Susa[1], Takayasu Mori[1], Soichiro Iimori[1], Shotaro Naito[1], Eisei Sohara[1], Shinichi Uchida[1], Kiyohide Fushimi[2], and Tatemitsu Rai[1]

[1]Department of Nephrology, Graduate School of Medical and Dental Sciences, Tokyo Medical and Dental University, 1-5-45 Yushima, Bunkyo, Tokyo 113-8519, Japan

[2]Department of Health Policy and Informatics, Graduate School of Medical and Dental Sciences, Tokyo Medical and Dental University, 1-5-45 Yushima, Bunkyo, Tokyo 113-8519, Japan

S Mandai and T Koide contributed equally.

Corresponding author: Shintaro Mandai

Department of Nephrology, Graduate School of Medical and Dental Sciences, Tokyo Medical and Dental University

1-5-45 Yushima, Bunkyo, Tokyo 113-8519, Japan

Tel: +81-3-5803-5214; Fax: +81-3-5803-5215; E-mail: smandai.kid@tmd.ac.jp

Figure S1. Patient flowchart.

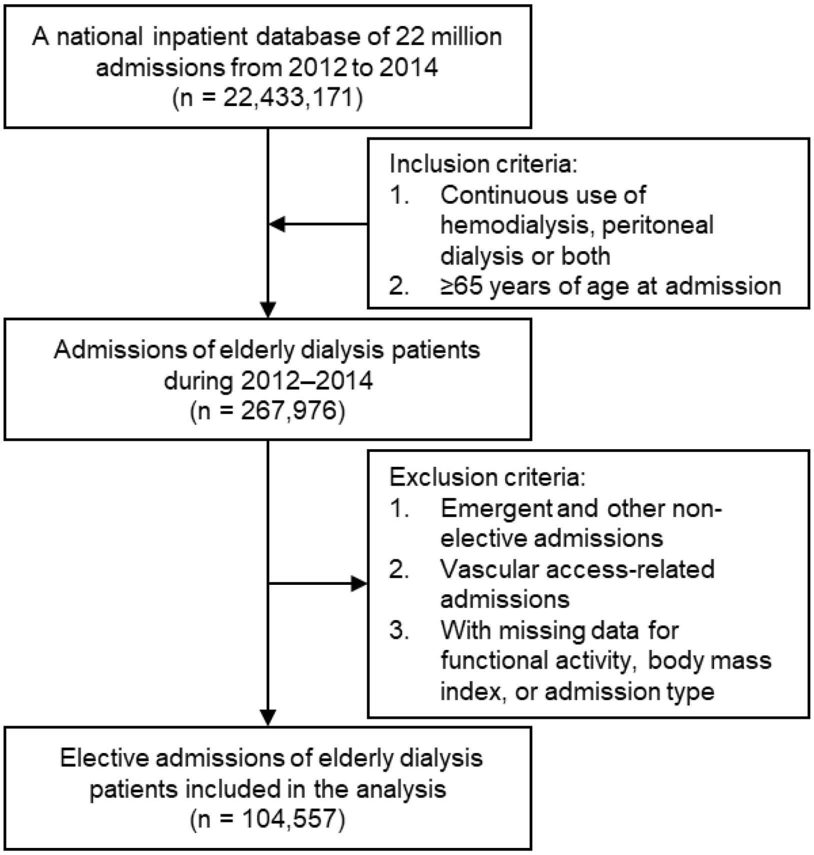

Figure S2. A risk matrix for in-hospital mortality according to admission functional status, age, and BMI categories among various clinical subgroups.

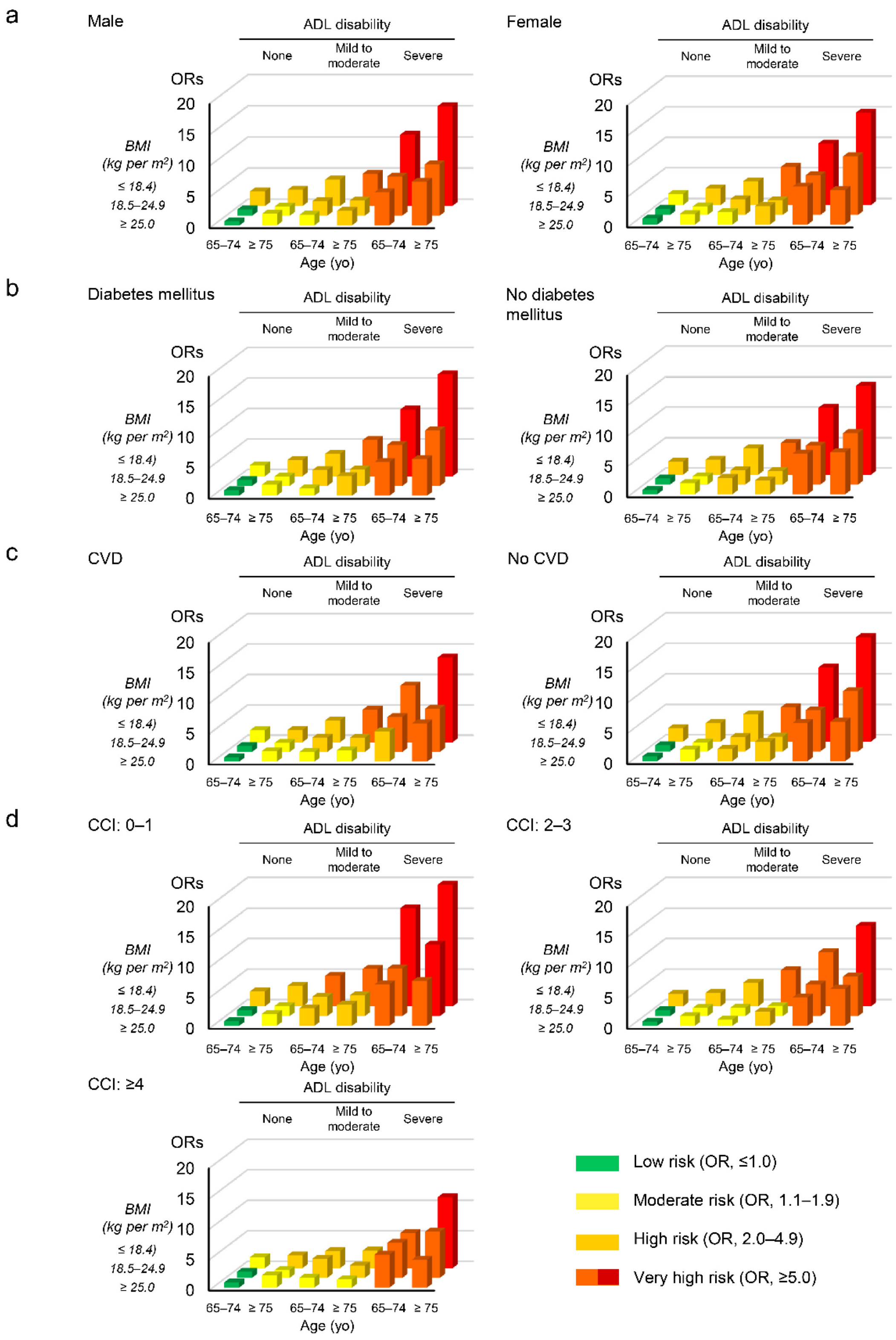

Multivariable logistic regression models were performed to assess the relationship between the risk stratification matrix based on functional status, age, and BMI categories and mortality risk according to various subgroups including sex (**a**), diabetes mellitus (**b**), CVD (**c**), and Charlson comorbidity index score (**d**). The odds ratio with 95% CI and numbers of events and individuals are described for each category. Models were adjusted for sex, dialysis modality, and Charlson comorbidity index. CVD was defined as any of myocardial infarction, congestive heart failure, peripheral vascular disease, or cerebrovascular disease.

ADL, activities of daily living; BMI, body mass index; CCI, Charlson comorbidity index; CVD, cardiovascular disease; OR, odds ratio

Table S1. Impact of requirement for assistance in feeding, transferring, going to toilet, or dressing on in-hospital mortality in elderly subjects on maintenance dialysis.

		OR (95% CI)	
Variable	No. of Events/N	Crude OR	Adjusted OR[a]
Disability with feeding	1,623/13,996	5.59 (5.22–5.98)	4.45 (4.14–4.77)
Disability with transferring	2,340/27,061	5.30 (4.95–5.67)	4.38 (4.08–4.70)
Disability with toileting	2,188/22,533	5.72 (5.35–6.12)	4.70 (4.38–5.04)
Disability with dressing	2,234/23,572	5.67 (5.30–6.07)	4.64 (4.32–4.98)
Disability with bathing	2,372/28,156	5.20 (4.85–5.57)	4.29 (3.99–4.60)

[a]Adjusted for age, sex, body mass index, dialysis modality, and Charlson comorbidity index. CI, confidence interval; OR, odds ratio

Table S2. Association between disability types and risk of higher hospital length of stay and medical cost in dialysis patients.

Category	No. of Events/N	OR (95% CI)	
		Crude OR	Adjusted OR[a]
Long length of stay (≥30 days)			
Disability with feeding	5,454/13,996	3.28 (3.15–3.40)	2.96 (2.84–3.08)
Disability with transferring	9,411/27,061	3.29 (3.18–3.39)	3.03 (2.93–3.14)
Disability with toileting	8,363/22,533	3.49 (3.38–3.61)	3.21 (3.11–3.33)
Disability with dressing	8,576/23,572	3.40 (3.29–3.52)	3.14 (3.03–3.25)
Disability with bathing	9,860/28,156	3.43 (3.32–3.54)	3.18 (3.08–3.29)
Highest quartile of medical cost			
Disability with feeding	4,369/13,996	1.43 (1.38–1.49)	1.40 (1.34–1.45)
Disability with transferring	8326/27,061	1.49 (1.44–1.54)	1.46 (1.41–1.51)
Disability with toileting	7,085/22,533	1.52 (1.47–1.57)	1.49 (1.44–1.54)
Disability with dressing	7,342/23,572	1.50 (1.45–1.55)	1.47 (1.42–1.52)
Disability with bathing	8,615/28,156	1.48 (1.44–1.53)	1.46 (1.41–1.50)

[a]Adjusted for age, sex, body mass index, dialysis modality, and Charlson comorbidity index. CI, confidence interval; OR, odds ratio
